# Danshen (*Salvia miltiorrhiza* Buge)–Gegen (*Pueraria lobata* (Willd.) Ohwi) Herb Pair Inhibits Ferroptosis After Ischemia–Reperfusion Injury Involving the Nrf2/System xc-/GPX4 Axis

**DOI:** 10.3390/antiox15070888

**Published:** 2026-07-17

**Authors:** Yin Liu, Yan Wang, Xinyu Shi, Ruomei Che, Xiaoli He

**Affiliations:** Institute of Medicinal Plant Development, Chinese Academy of Medical Sciences & Peking Union Medical College, Beijing 100193, China

**Keywords:** ischemic stroke, Danshen–Gegen herb pair, ferroptosis, lipid peroxidation, iron overload, Nrf2/system xc-/GPX4

## Abstract

**Background:** Danshen–Gegen is a classic herb pair in traditional Chinese medicine, which has been used to treat cardiovascular and cerebrovascular diseases. Ischemic stroke (IS) is a prevalent cerebrovascular condition; ferroptosis is one of the contributing factors driving the progression of IS. This study aims to determine the underlying mechanism and examine if Danshen–Gegen (DG) extract may prevent cerebral ischemia–reperfusion injury by preventing ferroptosis. **Methods:** The comprehensive compositional characterization of DG was analyzed by ultra-high-performance liquid chromatography coupled with hybrid quadrupole-orbitrap high-resolution mass spectrometry (UPLC-Q-orbitrap MS). The experiments were conducted in middle cerebral artery occlusion/reperfusion (MCAO/R) rats and oxygen-glucose deprivation/re-oxygenation (OGD/R) cells. The neuroprotective effects of DG on IS were assessed by examining rat survival rates, infarct volume, behavioral scores, and cerebral water content. Then, we tested the accumulation of Fe^2+^ and lipid peroxidation products such as reactive oxygen species (ROS), glutathione (GSH), malondialdehyde (MDA), myeloperoxidase (MPO), and 4-hydroxynonenal (4-HNE) in rats and cells. The expression of nuclear factor erythroid-derived 2-like 2 (Nrf2), Solute Carrier Family 7 Member 11 (-xCT), Glutathione peroxidase 4 (GPX4), Cyclooxygenase-2 (COX-2), Transferrin Receptor 1 (TFR1), and Long-chain-fatty-acid–CoA ligase 4 (ACSL4) was also assessed in vivo and in vitro. **Results:** UPLC-Q-orbitrap MS analysis was performed to characterize the chemical profile of DG, and a total of 33 chemical constituents were successfully identified. DG significantly alleviated the ischemic damage to brain tissue, reduced infarct volume, and improved neurological dysfunction. The content of Fe^2+^ and lipid peroxidation products was markedly decreased. Furthermore, DG could restore the expression of Nrf2, -xCT, and GPX4 with the inhibition of COX-2, TFR1, and ACSL4, thus achieving a suppressive effect on ferroptosis. **Conclusions:** The regulatory influence of DG via the Nrf2/System xc-/GPX4 axis may play a crucial role in alleviating ferroptosis and enhancing recovery from cerebral ischemia injury.

## 1. Introduction

Stroke is the second leading cause of disability and mortality in the world; it includes hemorrhagic stroke and ischemic stroke (IS), which account for approximately one-third of fatal diseases [1]. IS causes tissue softening due to insufficient blood supply, which results in neurological deficits [2]. This consequently results in significant cognitive and motor deficits [3]. Presently, only 11% of clinical patients with IS receive prompt treatment [4]. Therefore, it has been crucial to search for therapeutic drugs and methods.

As a distinct type of regulated cell death, ferroptosis is driven by iron-dependent lipid peroxidation [5]. This causes oxidative damage and loss of cell membrane function. The general mechanism of ferroptosis involves (1) metabolic pathways of iron, (2) amino acid metabolic pathways, and (3) lipid metabolic pathways [6]. Studies have demonstrated that heightened levels of lipid peroxidation products and elevated Fe^2+^ levels, which are two characteristic manifestations of ferroptosis, are observed in post-stroke patients. This observation suggests that ferroptosis may be regarded as a potential risk factor [7,8]. Numerous studies on protection against ischemia–reperfusion injury indicate that suppressing ferroptosis in ischemic brain tissue can ameliorate functional damage to tissues and neurons [9,10].

Danshen and Gegen are two common herbs in traditional Chinese medicine (TCM) that are frequently utilized to promote vasodilation, cardioprotection, and anti-atherosclerotic properties [11,12,13]. As a classic herb pair in TCM, Danshen–Gegen was first recorded in Shi Jinmo’s Herb Pairs, which has been extensively applied in various TCM formulas targeting ischemic stroke recently, such as Tongmai Granules and Dan-Deng-Tong-Nao Capsules [14]. Previous studies have confirmed that using the Danshen–Gegen herb pair could not only protect against cardiac IS injury [12,15], but also dilate the basilar arteries in rats [16]. Furthermore, puerarin and tanshinone IIA, derived from this herb pair, have been shown to mitigate neuronal damage following cerebral I/R injury [17]. Existing studies have confirmed that salvianolic acid A and puerarin inhibit ferroptosis by regulating the Nrf2 and p53 pathways, respectively, thus exerting protective effects against cerebral ischemic injury [18,19]. Despite their established benefits in the above-mentioned diseases, the neuroprotective mechanisms of DG in cerebral ischemia, particularly regarding ferroptosis regulation, remain unexplored. This study aims to investigate whether this herbal combination confers neuroprotection against cerebral ischemic injury by suppressing ferroptosis, and to further illustrate the underlying molecular mechanisms.

## 2. Materials and Methods

### 2.1. DG Herb Pair Extract

Danshen and Gegen (Tongrentang, Beijing, China) were extracted by the conventional heat reflux extraction method in a 2:1 ratio. The herb pair was added to a 10-fold volume of water and soaked at room temperature for 60 min. Then, reflux condensation extractions were conducted for 1 h each with 10-fold and 8-fold volumes of water, respectively [15,20]. The extracts underwent freeze-drying to yield DG powder, subsequently stored at −20 °C.

### 2.2. UPLC-Q-Orbitrap MS Analysis

#### 2.2.1. Preparation of Sample Solution

A total of 1.0 g of DG extract sample powder and 20 mL of 80% methanol were charged into a 50 mL centrifuge tube and ultrasonicated for 1 h. After that, 1 mL of the suspension was transferred into a 1.5 mL tube and centrifuged at 4 °C and 12,000 rpm for 10 min. Finally, 100 μL of supernatant was mixed with 100 μL of ultrapure water and then pipetted into an injection vial for detection.

#### 2.2.2. Chromatographic Conditions

A Vanquish Flex UPLC chromatograph (Thermo Fisher Scientific, Waltham, MA, USA) equipped with an ACQUITY UPLC HSS T3 column (2.1 mm (inner diameter) ×100 mm (length), 1.7 μm (particle dimension)) (Waters Corp., Milford, MA, USA) was used for separation. The mobile phase consisted of water (0.1% formic acid, phase A) and acetonitrile (phase B) with a flow rate of 0.3 mL/min, and the column temperature was 40 °C. The elution gradient is shown in Table 1, and the injection volume was 6.0 µL.

#### 2.2.3. MS Conditions

The MS data were collected by a hybrid quadrupole-orbitrap mass spectrometer (Q Exactive, Thermo Fisher Scientific, Waltham, MA, USA) equipped with a HESI-II spray probe. The parameters were set as follows: a positive ion source voltage of 3.7 kV and a negative ion source voltage of 3.5 kV, a heated capillary temperature of 320 °C, a sheath gas pressure of 30 psi, an auxiliary gas pressure of 10 psi, and a desolvation temperature of 300 °C. Both the sheath gas and the auxiliary gas were nitrogen. The collision gas was also nitrogen, with a pressure of 1.5 mTorr. The data were acquired in “Full scan/dd-MS2” mode. The parameters of the full scan were set as follows: resolution 70,000, auto gain control target 1 × 10^6^, maximum isolation time 50 ms, and *m*/*z* scan range 100–1500. The dd-MS2 data were collected with the parameters of resolution 17,500, auto gain control target 1 × 10^5^, maximum isolation time 50 ms, top *n* (*n* ≤ 10) most intense parent ions selected for fragmentation coupled with a dynamic exclusion mechanism, isolation window of *m*/*z*, collision energies 10 V, 30 V, and 60 V, and an intensity threshold 1 × 10^5^.

### 2.3. Animals

Six-week-old male Sprague Dawley rats (230 ± 10 g) were acquired from Beijing Vital River Laboratory Animal Technology Co., Ltd. (SCXK (Jing) 2021-0006, Beijing, China). Throughout the entire experimental period, rats were housed in specific pathogen-free (SPF) environments under a 12 h light/12 h dark cycle (temperature: 20–24 °C; relative humidity: 50 ± 10%), with optional standard provisions and unrestricted access to water. All experiments followed the regulations established by the Committee on Animal Ethics in Research at the Institute of Medicinal Plant Development (SLXD-20230725002, Beijing, China).

### 2.4. MCAO/R Model Construction

Following the administration of 2% pentobarbital sodium (1524001, Sigma-Aldrich, St. Louis, MO, USA) for anesthesia, animals were placed in the supine position on a temperature-controlled surgical table to expose the neck region. Throughout the surgical period, the neck vessels were revealed. The common carotid artery (CCA) and external carotid artery (ECA) were ligated, while the internal carotid artery (ICA) was temporarily clamped. A silicone nylon monofilament (2636-A4, Beijing Xinong, Beijing, China) was introduced from the CCA into the middle cerebral artery to induce ischemia for two hours [21]. Finally, the filament was withdrawn to restore blood flow, the vessels were ligated, and the incision was sutured. Rats in the sham group received an identical surgical procedure as the MCAO/R group except for filament insertion.

### 2.5. Animal Treatment Groups

A total of 160 rats were initially enrolled in this study. After excluding animals with failed model establishment, 30 rats were finally included in each group (*n* = 30 per group). All rats were divided randomly into five treatment groups: sham, model (MCAO/R), and three DG groups (100, 200, and 400 mg/kg). The initial dose was administered on the day of surgery, with subsequent daily treatment for 3 consecutive days. After all behavioral tests were finished, rats were sacrificed under deep anesthesia. Blood samples were collected via the abdominal aorta, and serum was isolated for subsequent experimental assays. After blood collection, the rats were decapitated, and brain tissues were harvested and stored at −80 °C for further analysis. Three rats from each group were randomly selected and perfused with 4% paraformaldehyde. Then, the brain tissues were immersed in 4% PFA at 4 °C for 24 h for subsequent pathological analysis.

### 2.6. Evaluation of Ischemia–Reperfusion (I/R) Injury

#### 2.6.1. Survival Rate and Content of Brain Water

Following surgery, brain injury was evaluated in all experimental animals, and the survival rate was calculated as the percentage of rats remaining alive throughout the entire experimental period [22]. At 72 h after reperfusion, rat brains were collected, weighed immediately to record the wet weight, and then dried at 105 °C for 48 h to determine the dry weight [23].

#### 2.6.2. Measurement of Infarct Volume

Brains were frozen at −20 °C for 15 min, sectioned into 2 mm coronal slices, and incubated with 1% 2,3,5-triphenyltetrazolium chloride (TTC, CT31153880, Coolaber, Beijing, China) at 37 °C for 30 min in the dark. Infarct volume and edema were measured from digital images using ImageJ (Version 1.53e, Bethesda, MD, USA [24].

#### 2.6.3. Modified Neurological Severity Score (mNSS)

Neurological deficits were assessed daily at 2 h after MCAO/R according to a Modified Neurological Severity Score (mNSS). All neurological scoring tests were performed using a blinded method, with the observers blinded to the treatments administered to the animals [22].

#### 2.6.4. Adhesive Removal Test

The rats were shifted to another quiet cage to adapt to the environment. After 5 min, the rats were captured, and a circular piece of sticky patch (diameter: 3 × 4 mm) was securely attached to the distal radial regions of each forelimb. Two adhesive paper stickers were attached. The time to remove each patch from the forelimbs was recorded, and the rats were then placed back into the quiet cage [25]. The rats were observed closely, and a timer was used to record the total time to remove the sticky patch from the left and right limbs from the time the rats perceived the patch and started trying to remove the sticky patch [26]. The assay was performed three times with intervals longer than 5 min.

### 2.7. Prussian Blue Staining

To evaluate cerebral iron deposition and the effect of DG on ferroptosis, a Prussian Blue Iron Stain Kit (G1029, Servicebio, Wuhan, China) was used for assessment. Following deparaffinization, the paraffin-embedded brain sections were stained for 1 h. They were then dehydrated, mounted, and counterstained with Nuclear Fast Red [27].

### 2.8. Immunohistochemistry (IHC)

Brain tissues were fixed, embedded in paraffin, and serially sectioned for subsequent IHC staining. After deparaffinization and antigen retrieval, paraffin sections were incubated with 5% goat serum at room temperature (RT) for 20 min to block non-specific binding. Then, they were incubated at 4 °C overnight with primary antibodies diluted 1:100 against GPX4 (GB154327, Servicebio, Wuhan, China), Nrf2 (GB113808, Servicebio, Wuhan, China), and ACSL4 (22401-1-AP, Proteintech, Wuhan, China). After washing, the sections were left to incubate in a dark environment with the HRP polymer (GB2304, Servicebio, Wuhan, China) for 30 min. Then, color development was carried out using the DAB mixture for 5 to 10 min. Ultimately, the nuclei were counterstained, and the slices were prepared for microscopic analysis. Three random fields of the ischemic penumbra were chosen for analysis [28,29].

### 2.9. Cell Culture

HT22 cells were cultivated under standard conditions (37 °C, 5% CO_2_) in high-glucose DMEM supplemented with 10% fetal bovine serum (FBS, Gibco, 16000044, Gibco, Grand Island, NY, USA) and 100 U/mL penicillin–streptomycin solution (Gibco, 15140122).

### 2.10. OGD/R Model and Drug Treatment

Cells were placed in DMEM medium without serum and glucose, and were cultured in a hypoxic incubator (Type III, COY Laboratory Products, Grass Lake, MI, USA) for 5 h, and then returned to be cultured under normal medium and conditions for another 12 h to establish the in vitro cerebral ischemia–reperfusion (IR) injury [30].

Cells in the OGD/R model, DG treatment groups, and ML385 group were grown in 200 µL/well of FBS-free glucose-free DMEM (2472342, Gibco, Grand Island, NY, USA), and exposed to oxygen-glucose deprivation for 5 h. After OGD/R, cells in the DG groups were incubated under normoxic conditions with serially diluted DG extract (3, 10, 30, 100, and 300 µg/mL crude drug) for an additional 12 h. The ML385 group was simultaneously treated with 100 µg/mL DG extract and 2 μM ML385 (M304758, Aladdin, Shanghai, China) for 12 h under a normoxic environment.

### 2.11. Evaluation of Cell Damage

#### 2.11.1. Cell Viability Assay

The cell viability was assessed using the MTT (917Q054, Solarbio, Beijing, China) test, with absorbance recorded at 490 nm via a microplate reader (Multiskan FC, Thermo Fisher Scientific, Waltham, MA, USA) [31].

#### 2.11.2. LDH Release Assay

Following the manufacturer’s instructions, the LDH detection kit (A020-2-2, Nanjing Jiancheng, Nanjing, China) was employed to ascertain the cellular lactate dehydrogenase (LDH) release volume [32].

#### 2.11.3. Cell Imaging

After a 12 h treatment, the medium was rinsed three times with PBS (8122214, Gibco, Grand Island, NY, USA). The cell morphology was observed and photographed by an inverted microscope (Ts2R, Nikon Eclipse, Tokyo, Japan) [26].

### 2.12. Cell Lipid ROS Assay

HT22 cells were treated with 5 µM BODIPY^TM^ 581/591 C11 (D3861, Thermo Fisher Scientific, Waltham, MA, USA) at 37 °C for 30 min after being rinsed twice with PBS. Then, they were imaged using a FluoView FV3000 confocal microscope (Ts2R, Nikon Eclipse, Tokyo, Japan). The cell fluorescence was measured by a BD AccuriTM C6 flow cytometer (Accuri C6, BD Biosciences, San Jose, CA, USA) and then analyzed with FlowJo software (Version 10.8.1; TreeStar, Ashland, OR, USA) [33].

### 2.13. Immunofluorescence (IF)

HT22 cells were fixed with 4% paraformaldehyde for 20 min, permeabilized with 0.5% Triton X-100 for 15 min, and blocked with 5% BSA for 2 h at 37 °C. The cells were incubated overnight (4 °C) with primary antibodies against Nrf2 and ACSL4, followed by Cy3-conjugated goat anti-rabbit IgG antibody (GB21303, Servicebio, Wuhan, China) and Cy3-conjugated goat anti-mouse IgG (GB21301, Servicebio, Wuhan, China) antibody for 1 h in the dark. Nuclei were counterstained with DAPI, and fluorescence images were obtained [34].

### 2.14. ELISA

The levels of IL-1β (SEKR-0002, Solarbio, Beijing, China), IL-6 (SEKR-0005, Solarbio, Beijing, China), and TNF-α (SEKR-0009, Solarbio, Beijing, China) in serum of rats and supernatants of HT22 cells were assessed by utilizing the corresponding ELISA kits according to the instruction manual [35].

### 2.15. MDA, GSH, and GSH/GSSG Contents

The contents of MDA and GSH were measured in rat serum and HT22 cells by using an MDA assay kit (A003-1-2, Nanjing Jiancheng, Nanjing, China) and a GSH assay kit (A006-2-1, Nanjing Jiancheng, Nanjing, China). A GSH/GSSG assay kit (S0053, Beyotime, Shanghai, China) was used to measure the ratio of reduced-to-oxidized GSH/GSSG in HT22 cells [28,36].

### 2.16. Transmission Electron Microscopy (TEM)

Peri-infarct cortical tissues (1 × 1 × 1 mm^3^) and HT22 cells were collected and fixed overnight in 2.5% glutaraldehyde phosphate buffer (P1126, Solarbio, Beijing, China), post-fixed in 1% osmium tetroxide (18456, Ted Pella Inc., Redding, CA, USA). The samples were then dehydrated, infiltrated in Araldite resin, and finally cut into 50–60 nm ultrathin sections for subsequent observation. A Hitachi H-7500 TEM (HT7800/HT7700, Hitachi, Tokyo, Japan) was used for imaging [37].

### 2.17. Detection of Fe^2+^

According to the iron colorimetric assay kit (BC5415, Solarbio, Beijing, China), the levels of Fe^2+^ in rat serum and HT22 cells were measured. Cell lysates were prepared by adding 500 µL of assay buffer and then centrifuging to obtain the supernatant. Subsequently, a standard curve was constructed using the reference standards in order to quantify the iron content [38].

### 2.18. MPO and 4-HNE Detection

MPO activity was measured using the MPO Peroxidation Activity Assay Kit (ab273334, Abcam, Cambridge, MA, USA) [33]. The intracellular 4-HNE (ab238538, Abcam, Cambridge, MA, USA) concentration was determined using the 4-HNE Assay Kit [39]. All samples, including rat serum and HT22 cells, were prepared according to the manufacturer’s instructions.

### 2.19. Western Blotting

Protein samples were lysed in RIPA buffer, separated by 10% SDS-PAGE (CW0022S, CWBIO, Beijing, China), and transferred onto Hybond-NC membranes (66485, PALL, Port Washington, NY, USA). After the membranes were blocked with 5% skim milk (D8340, Solarbio, Beijing, China) for 1 h at RT, primary antibodies against COX-2 (12375-1-AP, Proteintech, Wuhan, China, dilution ratio 1:1000), -xCT (ab307601, Abcam, Cambridge, MA, USA, dilution ratio 1:1000), TFR1 (ab269513, Abcam, Cambridge, MA, USA, dilution ratio 1:5000), and GAPDH (10494-1-AP, Proteintech, Wuhan, China, dilution ratio 1:5000) were then added and incubated overnight at 4 °C. After washing, the membranes were incubated with HRP-conjugated secondary antibodies for 1 h at RT. Protein bands were visualized using an enhanced chemiluminescence (ECL) substrate (P0018AS-1, Beyotime, Shanghai, China) and quantified with ImageJ software, with GAPDH serving as the loading control [40].

### 2.20. Statistical Analysis

GraphPad Prism (Version 10.1.2 San Diego, CA, USA) was used to analyze all of the data, and the results were displayed as mean ± standard deviation (SD). Survival curves were constructed via the Kaplan–Meier method for each group, and the log-rank test was conducted to test differences in survival between different groups. The data of the behavioral test were analyzed by two-way repeated-measures analysis of variance (ANOVA), followed by Tukey’s post hoc test for different time points. The other data were analyzed by one-way ANOVA followed by Tukey’s post hoc test. *p* < 0.05 was considered statistically significant.

## 3. Results

### 3.1. Identification of Chemical Constituents from DG

**Figure 1:**
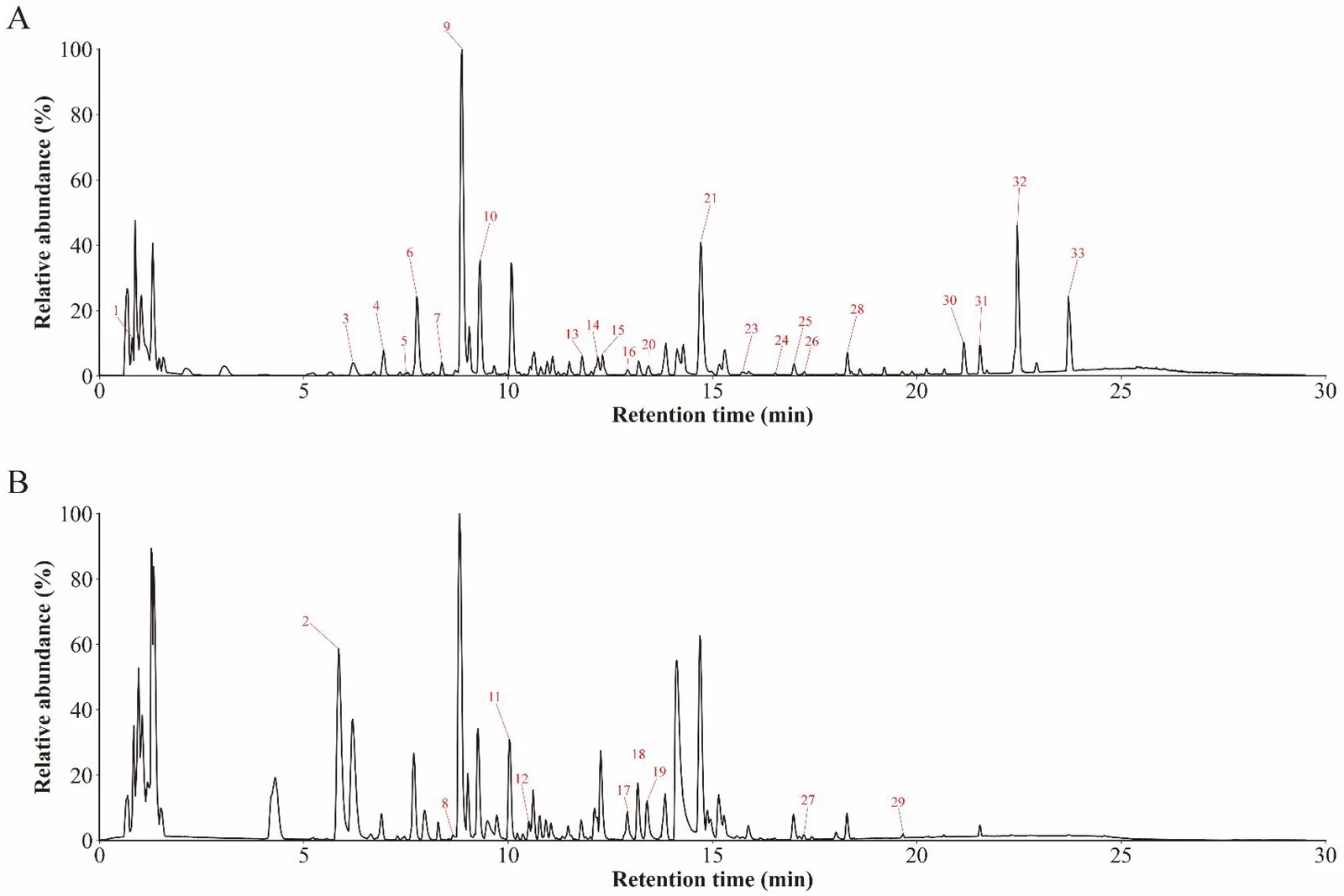
Detection of representative ingredients in DG by UPLC-Q-orbitrap MS. The base peak chromatogram in positive (**A**) and negative (**B**) ion mode. (The red numbers correspond to the serial numbers of compounds shown in Table 2).

The chemical constituents of the DG extract were analyzed using ultra-high-performance liquid chromatography coupled with quadrupole-orbitrap high-resolution mass spectrometry (UPLC-Q-orbitrap MS). The base peak ion (BPI) chromatograms, acquired in both positive and negative ionization modes, are presented in Figure 1A,B. Through systematic matching of the detected mass-to-charge ratio (*m*/*z*) values, as well as the characteristic information of the protonated molecular ion [M+H]^+^ and deprotonated molecular ion [M−H]^−^, against the reference substance database (TCM Pro 2.0) and theoretical database compiled from the literature and public databases, a total of 33 compounds were finally identified (Table 2).

The results showed that the chemical composition of DG extract was dominated by flavonoids, along with diverse classes of natural products, including phenolic acids, glycosides, and tanshinones. A total of 33 compounds were identified in this analysis, among which 15 were characteristic marker components of traditional Chinese medicine with well-defined pharmacological activities, with puerarin, salvianolic acid B, tanshinone IIA, and danshensu as the core representatives. These components are specific index ingredients of Gegen and Danshen, with confirmed cardiovascular and cerebrovascular protective effects, as well as antioxidant, anti-inflammatory, and lipid-regulating activities [41,42,43,44,45]. This study systematically clarified the chemical material basis of the DG extract, which provides a solid chemical foundation for subsequent research on its pharmacological activity mechanisms and the development of clinical applications.

### 3.2. Results of the In Vivo Experiment

#### 3.2.1. DG Ameliorates Cerebral Injury and Neurological Function in MCAO/R Rats

We examined the neuroprotective effects of DG in model rats by assessing the survival rate, neurological function (including mNSS and adhesive removal tests), brain water content, cerebral infarct volume, and hemispheric swelling in each group.

Compared with the sham group, rats in the MCAO model group showed a 27% decrease in survival rate and a 12.42% increase in brain water content, accompanied by extensive brain tissue necrosis and a cerebral infarction volume of 64.22%. These results confirmed that cerebral ischemia–reperfusion injury induces rat mortality, severe cerebral edema, and substantial brain tissue damage, verifying the stable and successful establishment of the animal model.

**Figure 2:**
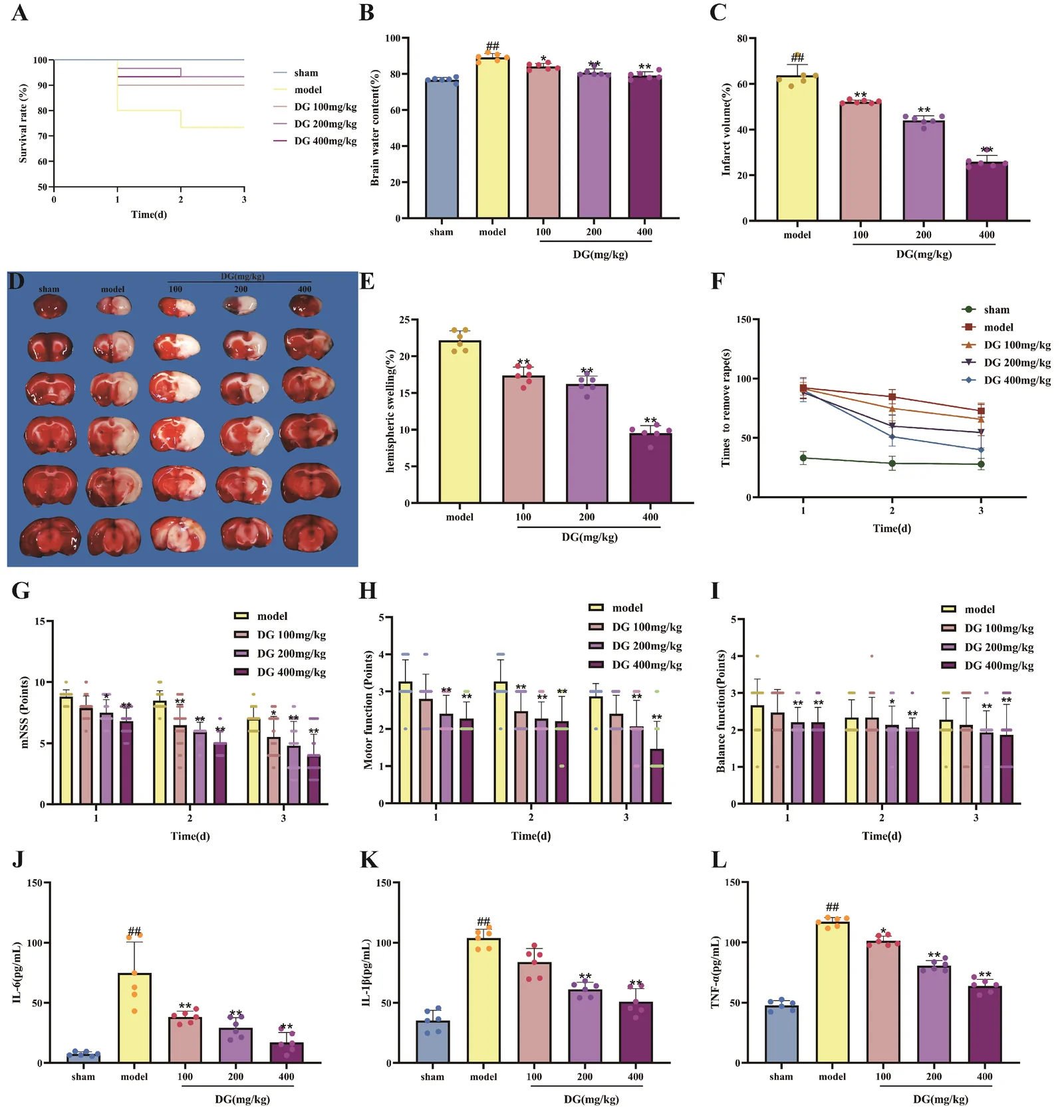
DG protects model rats after ischemic injury. (**A**) Effect of DG on the postoperative survival rate of MCAO/R rats (*n* = 30). (**B**) Effect of DG on the brain water content of MCAO/R rats (*n* = 6). (**C**) Effect of DG on the rate of infarct volume on the ischemic side of MCAO/R rats (*n* = 6). (**D**) TTC staining of the brain tissue (*n* = 6). (**E**) Effect of DG on the degree of hemispheric swelling on the ischemic side of MCAO/R rats (*n* = 6). (**F**) Effect of DG on the time to remove the adhesive tape in MCAO/R rats (*n* = 30). (**G**) Effect of DG on the postoperative mNSS score of MCAO/R rats (*n* = 30). (**H**) Effect of DG on the motor function and (**I**) balance function of MCAO/R rats (*n* = 30). (**J**–**L**) Effect of DG on the levels of IL-6, IL-1β, and TNF-α in the serum of MCAO/R rats (*n* = 6). (Data are presented as the mean ± SD. Note: ^##^ *p* < 0.01 vs. sham; * *p* < 0.05 vs. model; ** *p* < 0.01 vs. model).

The results demonstrated that DG administration at doses of 100, 200, and 400 mg/kg increased the survival rate of MCAO/R rats (Figure 2A) and reduced brain water content (Figure 2B). The TTC staining results indicated that DG markedly decreased the cerebral infarction volume, reduced hemisphere edema, and alleviated brain tissue damage (Figure 2C–E). The neurological deficit score indicates that DG can enhance neurological function, especially postoperative motor and balance abilities (Figure 2G–I). The adhesive removal test demonstrated that DG significantly shortened the adhesive patch removal time in MCAO/R rats compared to the model group (Figure 2F), indicating an improvement in sensorimotor deficit.

The levels of inflammatory cytokines were determined in rat serum. The ELISA results revealed that the levels of IL-6, IL-1β, and TNF-α were markedly increased in the model group relative to the sham group (*p* < 0.01). DG treatment dose-dependently inhibited the increase in three pro-inflammatory cytokines in MCAO/R rats. The 200 and 400 mg/kg DG groups significantly lowered all three inflammatory factors (*p* < 0.01) compared with the model group (Figure 2J–L). All these findings confirmed that DG exerts potent neuroprotective effects against cerebral I/R injury.

#### 3.2.2. Effect of DG on Ferroptosis in MCAO/R Rats

**Figure 3:**
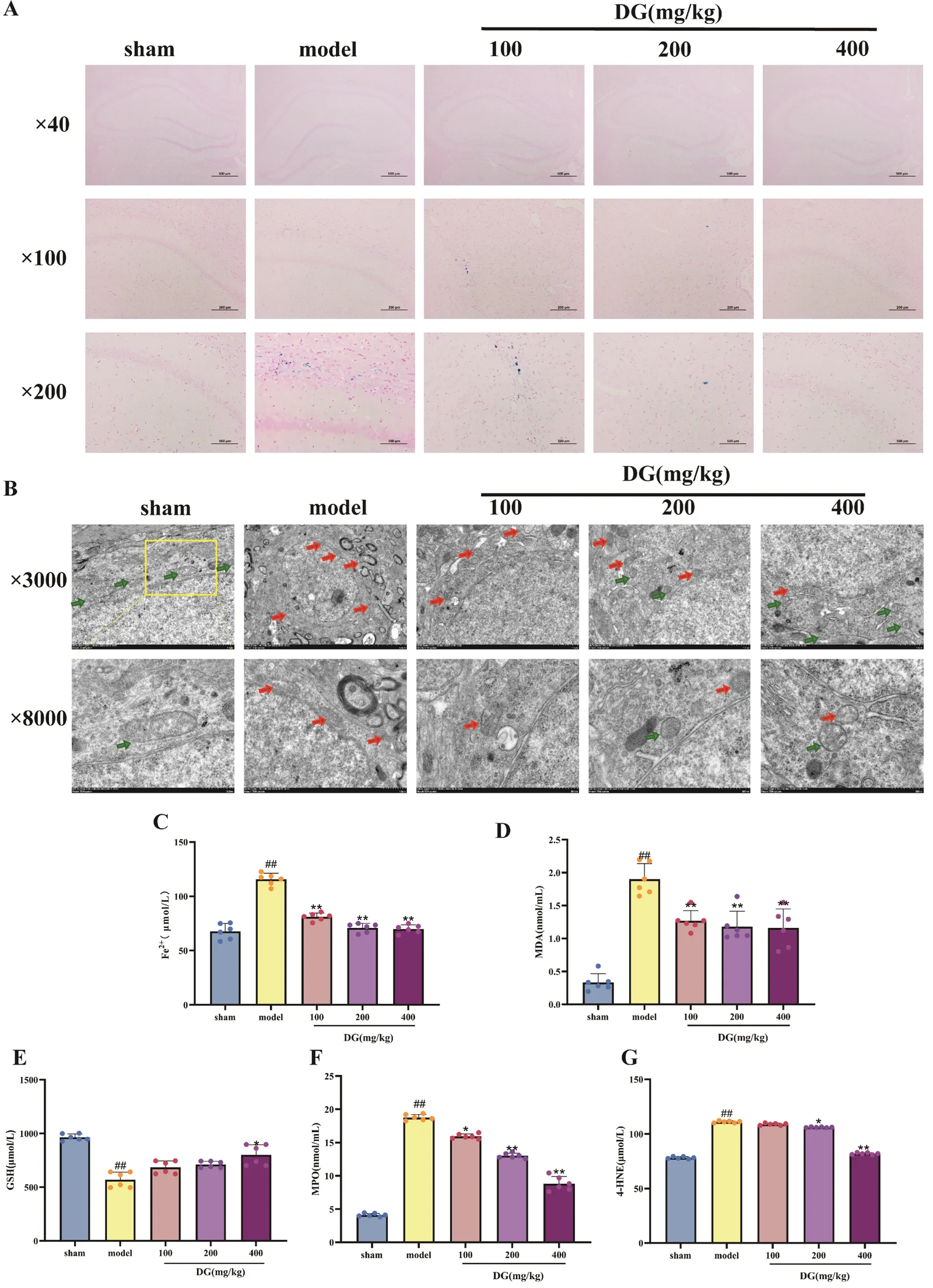
Effect of DG on ferroptosis in MCAO/R rats. (**A**) Result of Prussian Blue staining in the ischemic hippocampus and intercortical area on the ischemic side of rats after 3 days of treatment with DG (*n* = 3, Scale bar = 500, 200 or 100 µm). (**B**) Transmission electron microscopy of the mitochondrial structure in the ischemic penumbra of rats after 3 days of treatment with DG (*n* = 3, Scale bar = 5 or 1 µm). Note: Yellow boxes denote magnified areas; red arrows indicate mitochondria with structural damage, and green arrows represent normal mitochondria. (**C**) Effect of DG on the serum Fe^2+^ level in rats (*n* = 6). (**D**–**G**) Effect of DG on the levels of MDA, GSH, MPO, and 4-HNE in the serum of MCAO/R rats (*n* = 6). (Data are presented as the mean ± SD. Note: ^##^ *p* < 0.01 vs. sham; * *p* < 0.05 vs. model; ** *p* < 0.01 vs. model).

The deposition of Fe^2+^ in the ischemic brain tissue of model rats was observed. Iron accumulation in ischemic brain tissue was seen in the model group, but no such phenomena were detected in the sham group. The DG groups showed reduced Fe^2+^ deposition in the ischemic hippocampus and intercortical area on the ischemic side after MCAO/R (Figure 3A). Furthermore, the levels of Fe^2+^ in the serum of rats were also detected. The findings indicated that the MCAO/R injury resulted in an overload of Fe^2+^, whereas DG treatment led to a reduction in serum Fe^2+^ levels in rats (Figure 3C).

The results indicated that MCAO/R injury induced significant oxidative stress and ferroptosis, as demonstrated by a substantial elevation in lipid peroxidation products (MDA, 4-HNE) and the ferroptosis-related marker MPO, along with decreased antioxidant GSH content. DG not only diminished the concentration of lipid peroxidation products in model rats but also restored GSH homeostasis (Figure 3D–G).

We examined the mitochondrial ultrastructure in cells from the ischemic penumbra of I/R injury in the brain using TEM. In the model group, I/R injury induced rupture of the mitochondrial outer membrane, resulting in shrunken mitochondria. Meanwhile, the mitochondrial cristae were severely damaged, with disorganized structural integrity. These morphological changes were consistent with the characteristic manifestations of ferroptosis in cells, which indicated that ferroptosis occurred in I/R injury. DG alleviated the damage to the mitochondrial outer membrane, preserved the inner mitochondrial cristae, and restored the mitochondrial structure and shape in MCAO/R rats (Figure 3B).

These findings suggest that ferroptosis occurs after cerebral IR, and DG rescues this process through the regulation of lipid peroxidation.

#### 3.2.3. DG Inhibits Ferroptosis via the Nrf2/System xc-/GPX4 Axis in MCAO/R Rats

Studies have demonstrated that GPX4, Nrf2, and ACSL4 function as essential modulators of ferroptosis. GPX4 directly modulates glutathione equilibrium in glutamate metabolism [46], while Nrf2, a pivotal regulator of ferroptosis, is intricately linked to lipid peroxidation, inflammation, and oxidative stress [47]. Numerous studies indicate that ACSL4, a critical inducer of ferroptosis, mediates the conversion of PUFAs, which undergo lipid peroxidation to produce lethal lipid ROS under COX-2 regulation [48]. Transferrin receptor 1 (TFR1) is closely associated with the intracellular transport of Fe^3+^ in iron metabolism. The importance of the Nrf2/System xc-/GPX4 axis has been verified by regulating ferroptosis in cerebral I/R injury [49]. GPX4, Nrf2, and ACSL4 expression levels in the ischemic penumbra were detected by immunohistochemistry. TFR1, COX-2, and -xCT expression levels were quantitatively assessed by Western blotting (WB).

**Figure 4:**
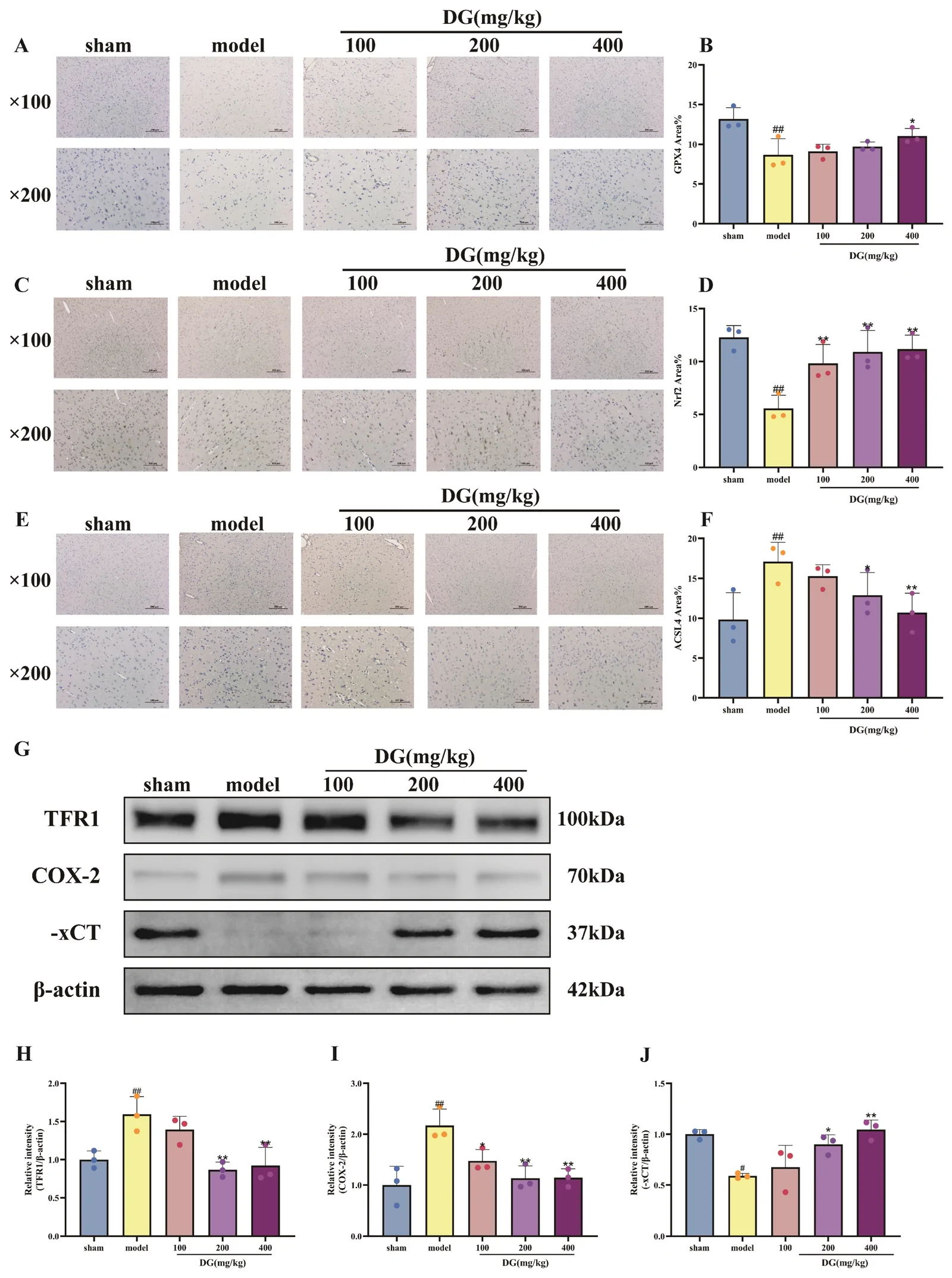
DG regulates the Nrf2/system xc-/GPX4 axis-mediated ferroptosis pathway. (**A**,**B**) The expressions of GPX4, (**C**,**D**) Nrf2, and (**E**,**F**) ACSL4 in the ischemic penumbra following three days of DG treatment (*n* = 3, Scale bar = 200 µm or 100 µm). (**G**–**J**) Impact of DG on TFR1, -xCT, and COX-2 in ischemic brain tissue following three days of treatment (*n* = 3). Positive protein staining is brown, and nuclei are counterstained blue. (Data are presented as the mean ± SD. Note: ^##^ *p* < 0.01 vs. sham; * *p* < 0.05 vs. model; ** *p* < 0.01 vs. model.) Original uncropped WB blots are provided in Figure S1.

The findings suggested that GPX4 and Nrf2 expressions were markedly diminished in the ischemic penumbra, while they were elevated in the DG groups, suggesting the protective impact of DG on ischemic tissue (Figure 4A–D). In contrast, ACSL4 expression was significantly increased, while it was decreased in DG groups (Figure 4E,F). Compared to the DG groups, the model group showed substantially greater levels of TFR1 and COX-2 expression, whereas -xCT expression was much lower than that in sham rats (Figure 4G–J). Overall, these results showed that DG exerts a regulatory effect on ferroptosis by reversing the abnormal alterations of the protein expressions in the Nrf2/System xc-/GPX4 axis.

### 3.3. Results of In Vitro Experiment

#### 3.3.1. Protection of DG on HT22 Cells with OGD/R Injury

**Figure 5:**
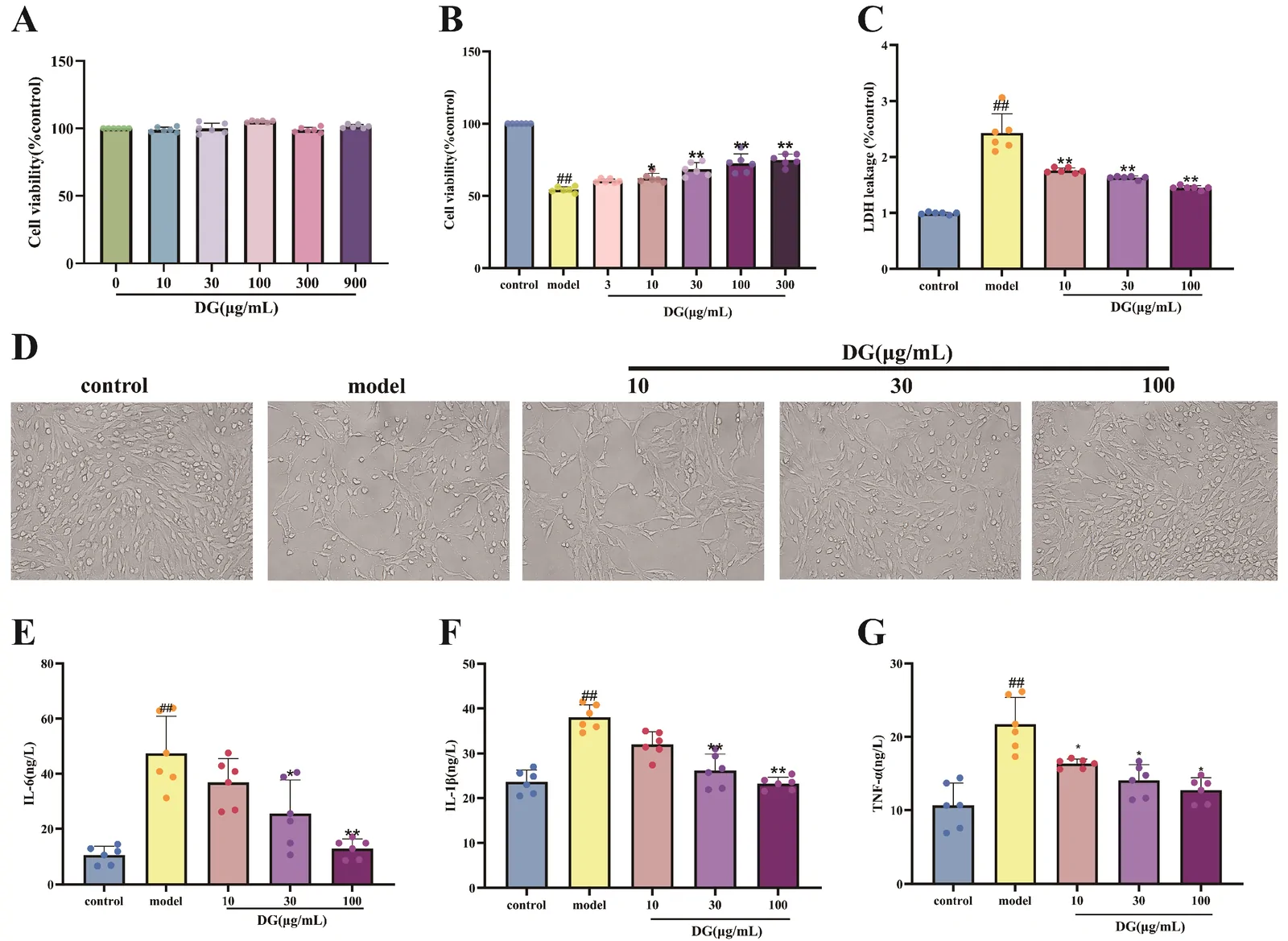
Protection of DG on HT22 cells with OGD/R injury. (**A**) The viability of HT22 cells with various doses of DG (*n* = 6). (**B**) The viability of OGD/R-injured HT22 cells with various doses of DG (*n* = 6). (**C**) The release of LDH (*n* = 6). (**D**) The morphology of HT22 cells subjected to OGD/R damage (inverted microscope, × 200) (*n* = 3). (**E**–**G**) The concentrations of IL-6, IL-1β, and TNF-α (*n* = 6). (Data are presented as the mean ± SD. Note: ^##^ *p* < 0.01 vs. control; * *p* < 0.05 vs. model; ** *p* < 0.01 vs. model).

First, cells given different concentrations of DG (10–900 μg/mL) showed no inhibitory effect on cell viability (Figure 5A). OGD/R-treated HT22 cells were administered DG at doses of 3–300 μg/mL. The results showed that DG improved viability and increased cell numbers, as well as gradually normalized cell morphology compared to the model group (Figure 5B,D). The model group also had a substantially greater LDH release rate in the supernatant compared to normal cells. However, the DG-treated groups showed a dose-dependent decrease compared to the model group (Figure 5C). OGD/R injury caused an apparent increase in the levels of IL-6, IL-1β, and TNF-α in HT22 cells. After treatment with DG, these levels were reduced to a certain extent (Figure 5E–G).

The results of these experiments indicated that DG could protect HT22 cells against OGD/R injury via increasing the survival rate, recovering cell morphology, decreasing the LDH release, and suppressing inflammation.

#### 3.3.2. Effect of DG on Ferroptosis in HT22 Cells with OGD/R Injury

**Figure 6:**
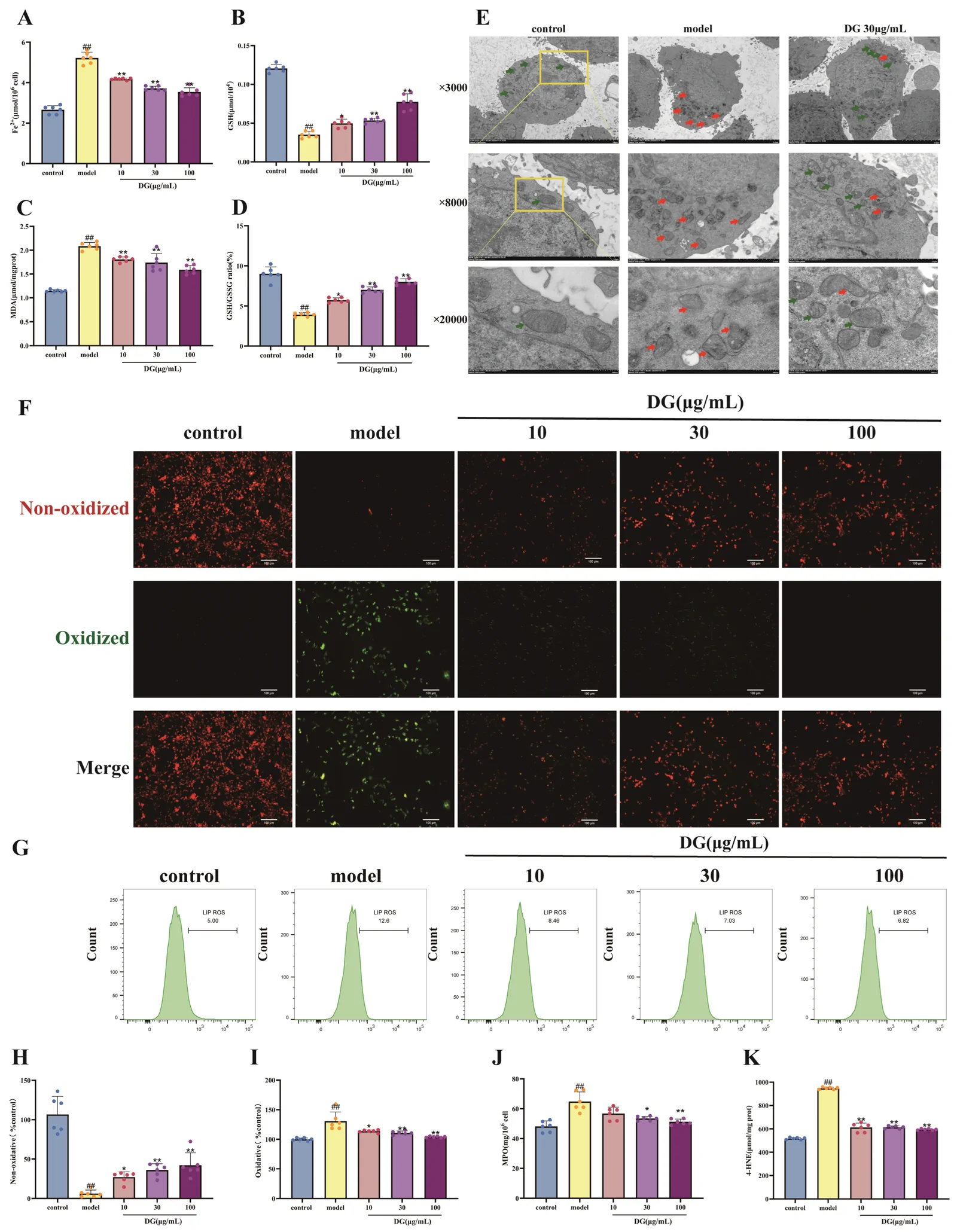
Effect of DG on ferroptosis in HT22 cells with OGD/R injury. (**A**–**D**) Effect of DG on the levels of Fe^2+^, GSH, MDA, and the rate of GSH/GSSG (*n* = 6). (**E**) Transmission electron microscopy of mitochondrial architecture in DG-treated HT22 cells subjected to OGD/R damage (*n* = 3, Scale bar = 5 or 2 µm, 500 nm). Note: green arrows represent normal mitochondria, red arrows indicate mitochondria with structural damage, and the yellow boxes indicate the magnified region. (**F**) Effect of DG on lipid ROS levels (*n* = 3). Fluorescence micrograph of cells with non-oxidized lipid ROS (red) and oxidized lipid ROS (green); magnification × 200. Scale bar = 100 µm. (**G**) Result of flow cytometry of cells with oxidized ROS (*n* = 3). (**H**,**I**) Effect of DG on the relative fluorescence intensity of non-oxidized and oxidized ROS related to lipid peroxidation (*n* = 6). (**J**,**K**) Effect of DG on the levels of MPO and 4-HNE (*n* = 6). (Data are presented as the mean ± SD. Note: ^##^ *p* < 0.01 vs. control; * *p* < 0.05 vs. model; ** *p* < 0.01 vs. model).

The findings demonstrated that DG markedly decreased the levels of Fe^2+^ and MDA, and increased the levels of GSH and the GSH/GSSG ratio in a dose-dependent trend compared with the model group (Figure 6A–D). In OGD/R-injured HT22 cells, mitochondria presented shortened and thickened morphology, accompanied by cell shrinkage, increased membrane density, and damaged inner cristae. DG alleviated OGD/R-induced mitochondrial morphological and ultrastructural damage (Figure 6E).

Intracellular lipid ROS was detected by BODIPY^TM^ 581/591 C11. The probe emits red fluorescence in the non-oxidized state and green fluorescence in the oxidized state, and the intensity of green fluorescence reflects the accumulation level of lipid ROS. The results showed that DG significantly suppressed the OGD/R-induced lipid ROS production in a dose-dependent manner (Figure 6F,H,I). Flow cytometric detection of oxidized ROS yielded identical results (Figure 6G). The model group had considerably higher levels of MPO and 4-HNE than those in the control group. In the DG groups (30 and 100 µg/mL), these concentrations dropped notably (Figure 6J,K).

Further analysis confirmed that DG alleviated OGD/R-induced ferroptosis in HT22 cells by downregulating ferroptosis-related indicators and mitigating lipid peroxidation.

#### 3.3.3. DG Inhibits Ferroptosis in OGD/R-Injured HT22 Cells Involving the Nrf2/System xc-/GPX4 Axis

To investigate whether DG inhibits ferroptosis by modulating this amino acid metabolic axis, we selected key proteins involved in the amino acid metabolic pathways for ferroptosis, such as -xCT, GPX4, and Nrf2, and performed immunofluorescence and Western blot analysis.

**Figure 7:**
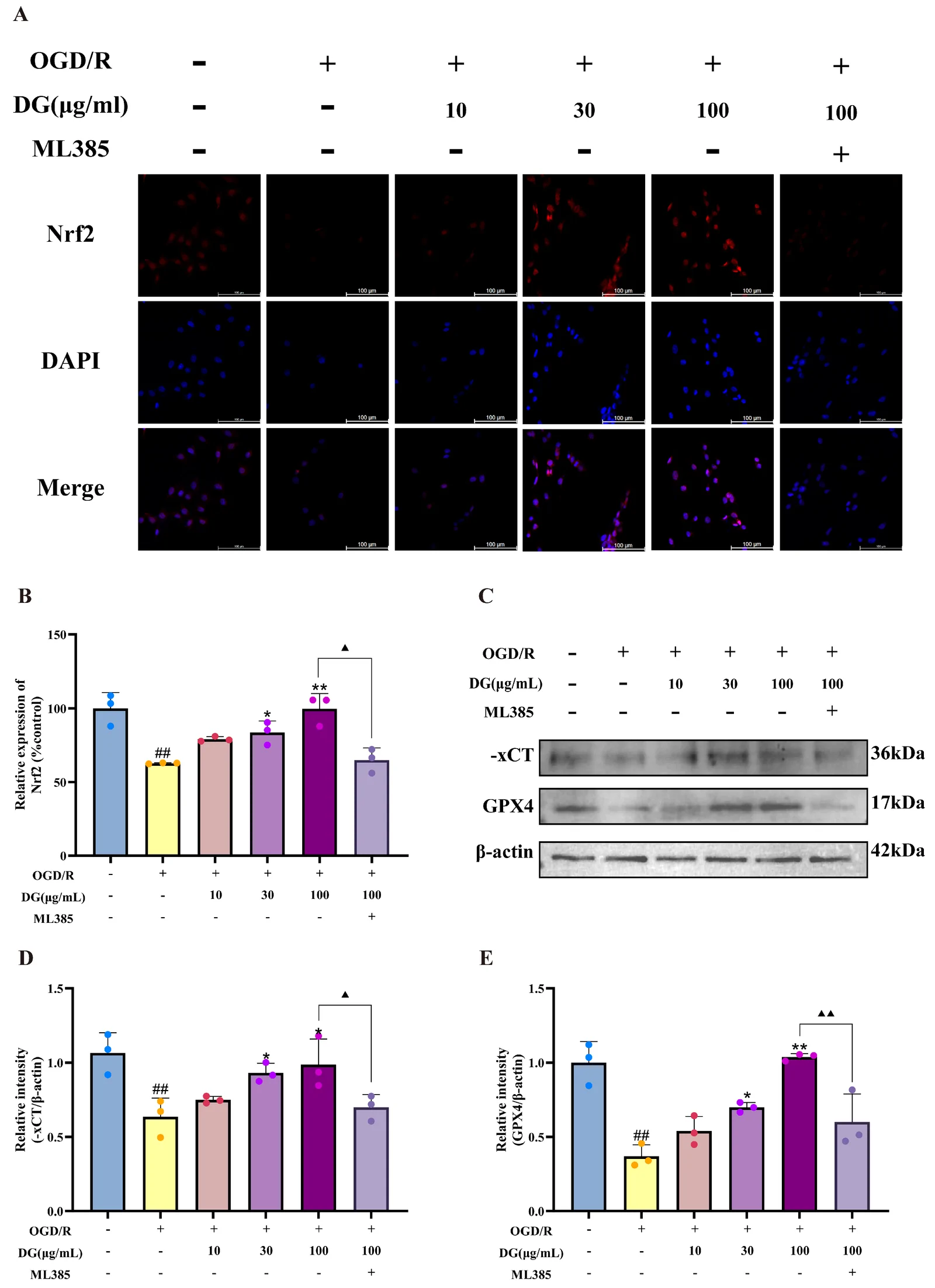
Regulatory effect of DG on the Nrf2/System xc-/GPX4 axis in OGD/R-injured HT22 cells. (**A**) Impact of DG on the fluorescence intensity of Nrf2 proteins related to ferroptosis (*n* = 3, Scale bar = 100 µm, Red: Nrf2; Blue: DAPI nuclear staining). (**B**) Quantification of Nrf2 protein expression (*n* = 3). (**C**) Impact of DG on -xCT and GPX4 protein expression associated with ferroptosis occurrence (*n* = 3). (**D**,**E**) Quantification of the -xCT and GPX4 levels (*n* = 3). (Data are presented as the mean ± SD. Note: ^##^ *p* < 0.01 vs. control; * *p* < 0.05 vs. model; ** *p* < 0.01 vs. Model; ^▲^ *p* < 0.05 vs. DG 100 μg/mL; ^▲▲^ *p* < 0.01 vs. DG 100 μg/mL.) Original uncropped WB blots are provided in Figure S2.

Immunofluorescence staining showed that the fluorescence intensity of Nrf2 was markedly decreased in HT22 cells of the OGD/R model group compared with the control group. After treatment with different concentrations of DG, the intracellular fluorescence intensity of Nrf2 was obviously recovered, and DG at concentrations of 30 μg/mL and 100 μg/mL significantly suppressed the OGD/R-induced loss of Nrf2 (Figure 7A,B). Combined intervention with the Nrf2-specific inhibitor ML385 significantly reversed the regulatory effect of DG on Nrf2 fluorescence intensity. Western blot analysis further confirmed that OGD/R injury induced abnormal downregulation of -xCT and GPX4 proteins, which could be effectively restored by DG treatment. However, co-treatment with ML385 reversed the regulatory effect of DG on the expression of -xCT and GPX4 (Figure 7C–E).

**Figure 8:**
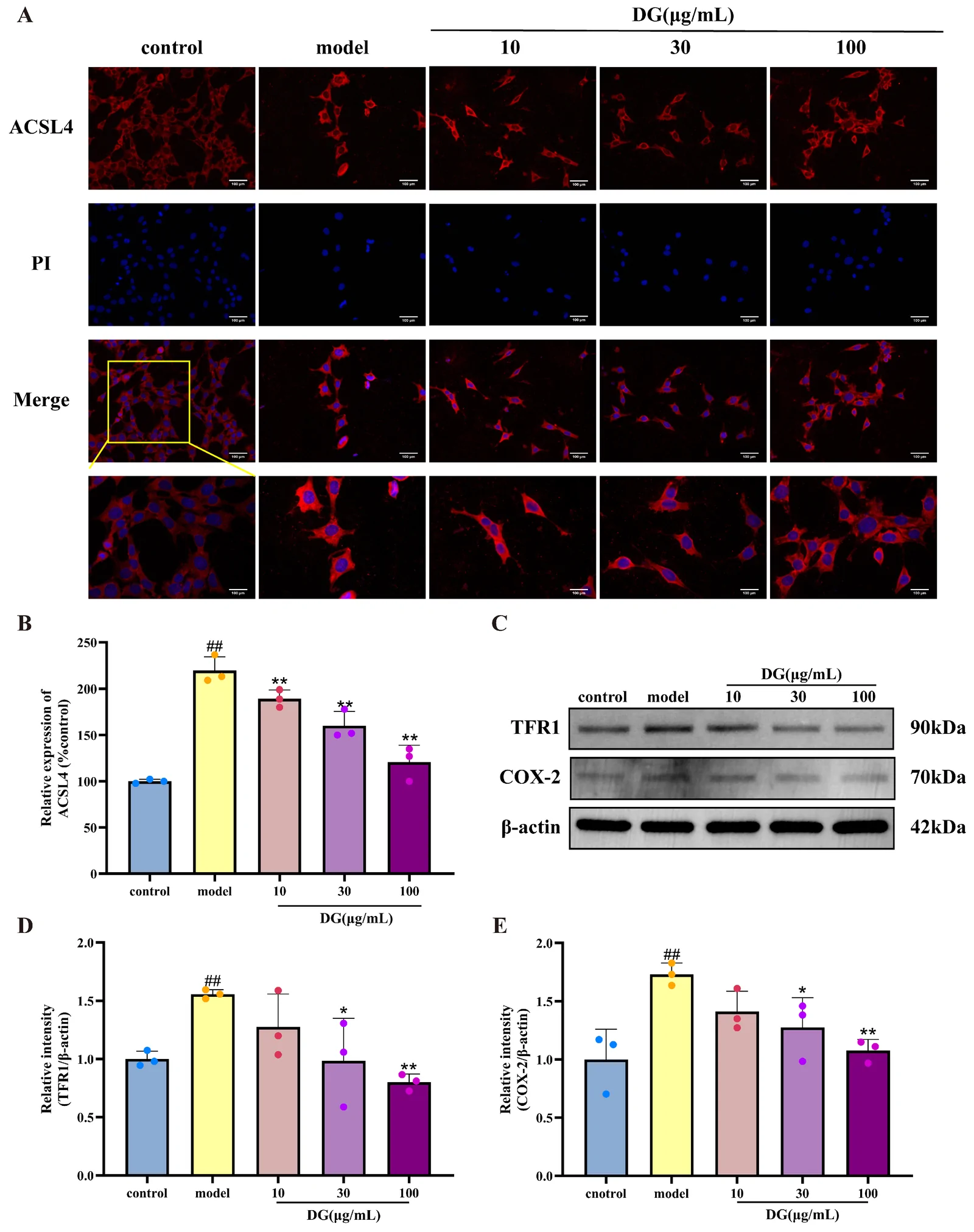
Regulatory effects of DG on the expression of proteins associated with ferroptosis. (**A**) Impact of DG on the fluorescence intensity of ACSL4 (*n* = 3, Scale bar = 100 or 50 µm, Red: ACSL4; Blue: DAPI nuclear staining. The yellow box indicates the magnified region). (**B**) Quantification of ACSL4 expression (*n* = 3). (**C**) Impact of DG on TFR1 and COX-2 expression associated with ferroptosis occurrence (*n* = 3). (**D**,**E**) Quantification of TFR1 and COX-2 levels (*n* = 3). (Data are presented as the mean ± SD. Note: ^##^ *p* < 0.01 vs. control; * *p* < 0.05 vs. model; ** *p* < 0.01 vs. model.) Original uncropped WB blots are provided in Figure S3.

As previously discussed, ACSL4, COX-2, and TFR1 are critically implicated in promoting ferroptosis through distinct mechanisms: TFR1 facilitates cellular iron uptake, ACSL4 enriches membranes with peroxidizable PUFAs, and COX-2 contributes to the generation of lipid mediators. The IF results showed that ACSL4 expression was elevated in the model group and significantly reduced in DG groups (Figure 8A,B). Western blot analysis showed that TFR1 and COX-2 protein expression levels were significantly increased in the model group, whereas the DG treatment markedly inhibited the OGD/R-induced elevation of their expression (Figure 8C–E).

In summary, DG may regulate the occurrence of ferroptosis in HT22 cells by modulating proteins related to Fe^2+^ transport and may prevent ferroptosis following cerebral ischemic injury involving the Nrf2/System xc-/GPX4 axis.

## 4. Discussion

Ischemic stroke is a devastating cerebrovascular disease characterized by high morbidity, mortality, and recurrence rates. In recent years, its incidence has increased significantly among younger populations. However, clinical treatment options for acute IS remain limited to mechanical thrombectomy and intravenous thrombolysis. The current clinical application is restricted by a narrow therapeutic time window, accompanied by multiple contraindications and negative sequelae such as post-thrombolysis hemorrhagic transformation [50]. Traditional Chinese medicine (TCM) has a long-standing and wide application in the treatment of ischemic stroke. It has been shown to alleviate post-stroke complications and optimize long-term prognosis through regulatory effects on multiple targets and signaling pathways [9,51]. Cerebral I/R injury can cause excessive accumulation of Fe^2+^, resulting in an increase in free radicals in neural cells and exacerbating nerve injury. Fe^2+^ overload is implicated in DNA damage, lipid peroxidation, neuronal apoptosis, and mitophagy in cerebral I/R injury, which further aggravates the pathological progression of I/R injury [52,53].

Danshen and Gegen are two classic medicinal herbs that are widely used in the management of cardiovascular and neurological disorders. Danshen can inhibit platelet activation in rats to reduce ischemic injury [54]. Its active components, such as tanshinone IIA, salvianolate, and dihydroisotanshinone I, have positive effects on ischemic brain injury and the maintenance of cellular iron balance [22,55,56]. As the water-soluble constituent of Danshen, salvianolic acid stabilizes mitochondrial function via the mtCx43-dependent PI3K/Akt pathway to counteract oxidative stress and neuronal damage triggered by cerebral ischemia–reperfusion [57]. The lipophilic component tanshinone IIA repairs damaged nerve fibers and alleviates focal cerebral ischemic injury by upregulating the expression of axonal regeneration markers neurofilament protein 200 and growth-associated protein-43 [58]. Gegen exerts neuroprotective effects against various central nervous system disorders [59,60,61]. It mitigates oxidative stress and ferroptosis via the AMPK/PGC1-α/Nrf2 pathway and suppresses ferroptosis following subarachnoid hemorrhage in rats [62,63]. Furthermore, puerarin, the primary bioactive constituent of Gegen, not only ameliorates cognitive and functional deficits in neurodegenerative diseases by modulating the expression levels of iron transporters [64,65], but also protects neurons to delay the progression of nervous system diseases via targeting the PI3K/Akt pathway [66]. All the above-mentioned active constituents were successfully identified through our UPLC-Q-orbitrap MS analysis. The neurotherapeutic potential of DG may be attributed to these bioactive ingredients.

Following cerebral I/R injury, blood–brain barrier (BBB) integrity is compromised, and neuronal iron uptake is significantly enhanced [67]. The phenomenon of iron deposition stems from impaired cellular antioxidant defense systems. Intracellular Fe^2+^ enters mitochondria through mitochondrial iron transporters, disrupting electron transport chain function and leading to excessive H_2_O_2_ production. Furthermore, excess Fe^2+^ reacts with H_2_O_2_ via the Fenton reaction, generating toxic ROS that induce lipid peroxidation and membrane damage. This cascade ultimately culminates in ferroptosis [68,69,70].

Lipid peroxidation is the core hallmark and driving event of ferroptosis [71], and is also a key pathological link leading to neuronal damage after cerebral I/R injury [72]. Uncontrolled accumulation of lipid ROS and toxic peroxidation end products can directly damage neuronal cell membranes and mitochondria, disrupt intracellular redox homeostasis, and ultimately induce irreversible neuronal death [73], which has become a promising therapeutic target for ischemic stroke. In this study, we found that DG remarkably ameliorated cerebral IR injury both in vitro and in vivo. DG treatment markedly attenuated the OGD/R and MCAO-induced elevation of classic lipid peroxidation markers, including MDA, 4-HNE, and MPO, and suppressed excessive lipid ROS accumulation both in vitro and in vivo. This effect was accompanied by the alleviation of Fe^2+^ overload, restoration of GSH/GSSG balance, and improvement in mitochondrial morphological damage. Furthermore, the study of iron homeostasis should not only focus on iron content but also analyze the expression levels of proteins associated with iron transport such as TFR1 [74]. It has been reported that neurons absorb iron via TFR1 and DMT1, and cerebral I/R injury significantly elevates the expression of TFR1 and DMT1, thereby aggravating intracellular iron overload [75]. Meanwhile, TFR1 has been clearly identified as a specific ferroptosis marker, and its upregulation is directly linked to the activation of the ferroptosis pathway [76]. In our study, DG significantly reduced the abnormal upregulation of TFR1 induced by cerebral ischemia–reperfusion injury and attenuated intracellular Fe^2+^ overload by inhibiting iron uptake.

The amino acid metabolism pathway in ferroptosis primarily centers on system xc-, a cystine/glutamate antiporter situated on the cell membrane. As a heterodimeric protein complex composed of the light-chain functional subunit -xCT and the heavy-chain subunit SLC3A2, system xc- is responsible for transporting extracellular cystine into the cytoplasm [77,78]. Internalized cystine is subsequently reduced to cysteine, the rate-limiting substrate for GSH biosynthesis. As a core endogenous antioxidant, GSH serves as an indispensable cofactor for the catalytic function of GPX4 [79]. GPX4 specifically catalyzes the reduction of phospholipid hydroperoxides, and is therefore essential for maintaining plasma membrane lipid integrity, blocking the cascade amplification of lipid peroxidation, and preventing ferroptotic cell death [80]. The transcription factor Nrf2 acts as a key upstream regulator of this entire pathway, coordinating the expression of multiple target genes involved in cystine uptake, GSH synthesis, and GPX4 expression to exert transcriptional control over ferroptosis progression [81].

Mechanistically, the inhibitory effect of DG on lipid peroxidation and ferroptosis is closely mediated by the activation of the Nrf2/System xc-/GPX4 signaling axis. Specifically, DG blocks the cascade amplification of lipid peroxidation and subsequent neuronal ferroptosis, and enhances the expression of GPX4, the key rate-limiting enzyme for the clearance of toxic lipid hydroperoxides. Meanwhile, DG activates the Nrf2 signaling pathway to rescue the decreased expression of -xCT, which ensures adequate intracellular GSH biosynthesis to sustain the antioxidant function of GPX4. To fully verify this underlying mechanism, we further performed experiments using ML385, a specific inhibitor of Nrf2. ML385 pretreatment significantly reversed the regulatory effects of DG on OGD/R-suppressed -xCT and GPX4 expression. These findings further confirm that the anti-ferroptotic effects of DG are dependent on the Nrf2/System xc-/GPX4 pathway. Consistent with our findings, previous studies have confirmed that the main active components of DG exert significant antioxidant and neuroprotective effects [57,82,83]. Our study further extends these findings: the anti-ferroptosis mechanism of DG is intricately linked to the three canonical ferroptosis regulatory pathways, among which the Nrf2/System xc-/GPX4 axis serves as the core regulatory cascade. These findings not only fill the gap in the research on the neuroprotective mechanism of the Danshen–Gegen herb pair, but also provide a solid theoretical basis for its clinical application in the treatment of ischemic stroke. Nevertheless, further in-depth validation experiments are warranted to consolidate the conclusions of this study.

In summary, this study demonstrates that DG alleviates ferroptosis-associated cellular damage, characterized by Fe^2+^ accumulation and mitochondrial dysfunction. The protective effect is attributed to a reduction in lipid peroxidation. Further mechanistic studies demonstrated that the Nrf2/System xc-/GPX4 antioxidant axis serves as an important mediator underlying DG’s neuroprotective effects against cerebral I/R injury.

## 5. Conclusions

**Figure 9:**
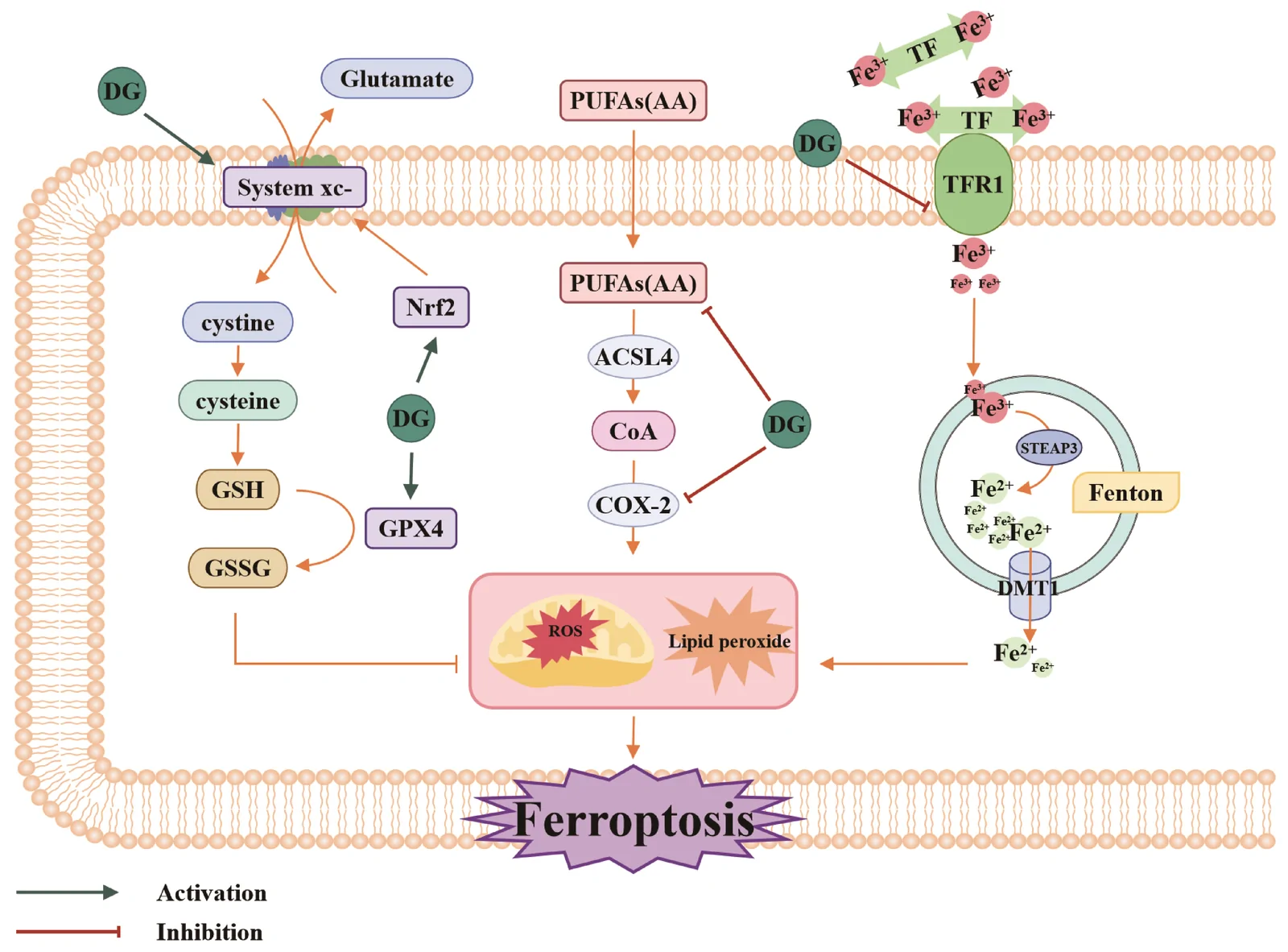
Mechanism diagram of DG inhibiting ferroptosis after cerebral ischemic injury involving the Nrf2/System xc-/GPX4 axis (Created in biorender.com). GSSG, synthesized oxidized glutathione; GSH, glutathione; Nrf2, Nuclearfactor erythroidderived 2-like 2; GPX4, Glutathione peroxidase 4; PUFAs, Polyunsaturated Fatty Acids; AA, Arachidonic Acid; ACSL4, Long-chain-fatty-acid–CoA ligase 4; CoA, Coenzyme A; ROS, reactive oxygen species; TFR1, Transferrin Receptor 1; TF, Transferrin; STEAP3, Six-Transmembrane Epithelial Antigen of Prostate 3; DMT1, Divalent Metal Transporter 1.

DG exerts neuroprotective and therapeutic effects against cerebral I/R injury by inhibiting ferroptosis (Figure 9). These findings provide novel insights into ischemic stroke therapy.

## Figures and Tables

**Table 1:** Elution gradient.

Time (min)	Mobile Phase	
	A (v%)	B (v%)
0	98	2
1.0	98	2
14.0	70	30
25.0	0	100
28.0	0	100
28.1	98	2
29.5	98	2

**Table 2:** The identification results of chemical components in DG extract.

Peak	RT (min)	Compound	Formula	Experimental *m*/*z*	Theoretical *m*/*z*	Adduct	Fragments	Error (ppm)
1	0.85	Verbascose	C_30_H_52_O_26_	829.2813225	829.2820	[M + H]^+^	85.0296, 163.0602, 91.0400, 61.0299, 145.0497, 127.0394, 325.1129	−0.77
2	4.30	Danshensu	C_9_H_10_O_5_	197.0446	197.0455	[M − H]^−^	72.9905, 135.0430, 179.0334, 123.0428, 197.0443	−4.57
3	6.22	Protocatechualdehyde	C_7_H_6_O_3_	139.0388561	139.0390	[M + H]^+^	111.0447, 93.0345, 65.0400, 139.0392	−0.83
4	6.98	Hypaphorine	C_14_H_18_N_2_O_2_	247.143677	247.1441	[M + H]^+^	188.0707, 146.0602, 60.0823, 118.0656, 144.0810	−1.74
5	7.47	Puerarin 4′-O-glucoside	C_27_H_30_O_14_	601.1524375	601.1528	[M + Na]^+^	601.1534, 439.1007, 420.1272, 66.8480	−0.59
6	7.76	3′-Hydroxypuerarin	C_21_H_20_O_10_	433.1121226	433.1129	[M + H]^+^	433.1135, 313.0707, 283.0602, 397.0924, 415.1028, 337.0707, 367.0816	−1.85
7	8.37	Apigenin 6-C-glucoside 8-C-arabinoside	C_26_H_28_O_14_	565.1545409	565.1552	[M + H]^+^	565.1558, 313.0706, 283.0600, 433.1132, 415.1025, 397.0922, 337.0706	−1.14
8	8.62	Mirificin	C_26_H_28_O_13_	547.1461543	547.1457	[M − H]^−^	267.0666, 547.1468, 295.0612, 152.0094, 108.0192, 277.0505, 59.0112	0.80
9	8.87	Puerarin	C_21_H_20_O_9_	417.117082	417.1180	[M + H]^+^	417.1184, 297.0758, 267.0652, 268.0719, 381.0970, 351.0865, 399.1078	−2.23
10	9.31	3′-Methoxypuerarin	C_22_H_22_O_10_	447.1278079	447.1286	[M + H]^+^	447.1292, 327.0864, 297.0759, 411.1077, 298.0822, 429.1192, 381.0973	−1.72
11	10.03	Daidzin	C_21_H_20_O_9_	461.1090096	461.1089	[M + FA − H]^−^	253.0502, 415.1039, 252.0426, 461.1096	0.18
12	10.50	Vitexin	C_21_H_20_O_10_	431.0981684	431.0984	[M − H]^−^	311.0566, 431.0988, 283.0613, 135.0427	−0.47
13	11.81	Genistin	C_21_H_20_O_10_	433.1123108	433.1129	[M + H]^+^	271.0602, 433.1130, 153.0184, 215.0703	−1.42
14	12.29	Salvianolic acid D	C_20_H_18_O_10_	436.1230975	436.1238	[M + NH_4_]^+^	221.0445, 159.0442, 131.0494, 177.0547, 147.0442, 175.0391, 103.0550	−1.73
15	12.31	Kushenol O	C_27_H_30_O_13_	563.1748647	563.1759	[M + H]^+^	563.1771, 311.0914, 281.0810, 413.1240, 431.1342, 395.1135, 365.1016	−1.87
16	12.88	Malonyldaidzin	C_24_H_22_O_12_	503.1179461	503.1184	[M + H]^+^	255.0651, 503.1192, 85.0295, 199.0755	−0.91
17	12.92	Salvianolic acid B	C_36_H_30_O_16_	717.1469551	717.1461	[M − H]^−^	321.0413, 519.0925, 109.0271, 339.0517, 185.0234, 295.0612, 279.0294	1.18
18	13.18	Rosmarinic acid	C_18_H_16_O_8_	359.0772604	359.0772	[M − H]^−^	161.0226, 197.0443, 359.0778, 72.9905, 135.0429, 133.0273, 179.0335	0.05
19	13.41	Lithospermic acid	C_27_H_22_O_12_	537.1037404	537.1038	[M − H]^−^	109.0271, 185.0228, 295.0611, 493.1137, 135.0429, 72.9904, 159.0427	−0.20
20	13.41	Salvianolic acid F	C_17_H_14_O_6_	297.0752088	297.0757	[M + H − H_2_O]^+^	279.0652, 297.0758, 251.0705, 233.0598, 205.0649, 223.0757, 261.0551	−1.72
21	14.71	Daidzein	C_15_H_10_O_4_	255.0645073	255.0652	[M + H]^+^	255.0653, 199.0756, 137.0235, 227.0706	−2.74
22	14.86	Monomethyl lithospermate	C_28_H_24_O_12_	551.119614	551.1195	[M − H]^−^	135.0429, 197.0442, 309.0771, 507.1298, 72.9904, 551.1212, 294.0540	0.21
23	15.76	Calycosin	C_16_H_12_O_5_	285.0751885	285.0757	[M + H]^+^	285.0759, 270.0522, 253.0496, 225.0549	−1.98
24	16.52	Tanshindiol A	C_18_H_16_O_5_	313.1063722	313.1070	[M + H]^+^	267.1019, 221.0949, 295.0970, 249.0915, 313.1068, 107.0499, 253.0860	−2.17
25	17.00	Genistein	C_15_H_10_O_5_	271.0594289	271.0601	[M + H]^+^	271.0602, 153.0184, 215.0706, 243.0654	−2.48
26	17.25	Przewaquinone E	C_18_H_16_O_5_	313.1063028	313.1070	[M + H]^+^	107.0499, 267.1016, 313.1064, 253.0858, 295.0959	−2.39
27	17.33	Tectorigenin	C_16_H_12_O_6_	299.0560987	299.0561	[M − H]^−^	299.0561, 284.0329, 255.0306, 227.0340	−0.04
28	18.30	Formononetin	C_16_H_12_O_4_	269.0801668	269.0808	[M + H]^+^	269.0809, 254.0560, 213.0911, 237.0550	−2.49
29	19.66	Biochanin A	C_16_H_12_O_5_	283.0610774	283.0612	[M − H]^−^	283.0613, 268.0379, 267.0293, 239.0352	−0.42
30	21.14	Dihydrotanshinone I	C_18_H_14_O_3_	279.1009783	279.1016	[M + H]^+^	279.1018, 261.0908, 233.0967, 205.1022, 149.0236, 190.0777, 169.0649	−2.13
31	21.54	Neocryptotanshinone	C_19_H_22_O_4_	315.1581604	315.1591	[M + H]^+^	297.1487, 315.1593, 279.1384, 254.0941, 251.1436	−2.95
32	22.46	Cryptotanshinone	C_19_H_20_O_3_	297.1478236	297.1485	[M + H]^+^	297.1486, 251.1431, 254.0937, 279.1385	−2.36
33	23.71	Tanshinone IIA	C_19_H_18_O_3_	295.1323864	295.1329	[M + H]^+^	295.1328, 277.1225, 249.1268, 252.0785	−1.65

## Data Availability

The original contributions presented in this study are included in the article/Supplementary Material. Further inquiries can be directed to the corresponding author.

## Supplementary Materials

The following supporting information can be downloaded at: https://www.mdpi.com/article/10.3390/antiox15070888/s1, Figure S1: The original image corresponding to Figure 4G in the main text; Figure S2: The original image corresponding to Figure 7C in the main text; Figure S3: The original image corresponding to Figure 8C in the main text.

## Abbreviations

The following abbreviations are used in this manuscript:

IS	Ischemic stroke
Danshen	*Salvia miltiorrhiza* Buge
Gegen	*Pueraria lobata* (Willd.) Ohwi
DG	Danshen–Gegen
MCAO/R	Middle cerebral artery occlusion/reperfusion
OGD/R	Oxygen glucose deprivation/re-oxygenation
ROS	Reactive oxygen species
GSH	Glutathione
MDA	Malondialdehyde
MPO	Myeloperoxidase
4-HNE	4-hydroxynonenal
PUFAs	Polyunsaturated Fatty Acids
AA	Arachidonic Acid
GSSG	Synthesized oxidized glutathione
CoA	Coenzyme
Nrf2	Nuclear factor erythroid-derived 2-like 2
-xCT (or SLC7A11)	Solute Carrier Family 7 Member 11
GPX4	Glutathione peroxidase 4
COX-2	Cyclooxygenase-2
TFR1	Transferrin Receptor 1
TF	Transferrin
STEAP3	Six-Transmembrane Epithelial Antigen of Prostate 3
ACSL4	Long-chain-fatty-acid--CoA ligase 4
DMT1	Divalent Metal Transporter 1
System xc-	Cystine/glutamate antiporter system (specific subunit -xCT)
TCM	Traditional Chinese Medicine
CCA	Common carotid artery
ECA	External carotid artery
ICA	Internal carotid artery
I/R	Ischemia–reperfusion
