# Supplementary material for: Danshen (Salvia miltiorrhiza Buge)–Gegen (Pueraria lobata (Willd.) Ohwi) Herb Pair Inhibits Ferroptosis After Ischemia–Reperfusion Injury Involving the Nrf2/System xc-/GPX4 Axis
Source: Antioxidants (Basel). 2026 Jul 17;15(7):888. doi: 10.3390/antiox15070888 (PMC13404947; doi:10.3390/antiox15070888)
Supplement: Supplementary file 1 [file antioxidants-15-00888-s001.zip › antioxidants-4337362-Supplementary Materials.pdf]

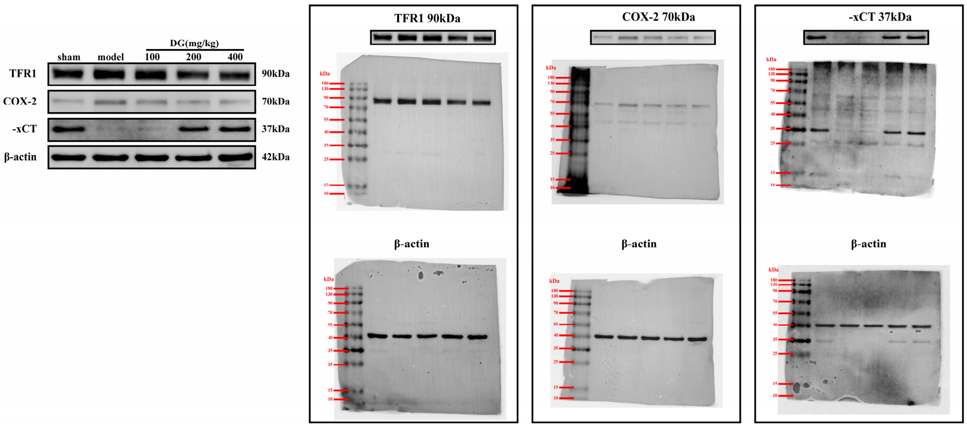

Figure S1. The original image corresponding to Figure 4G in the main text.

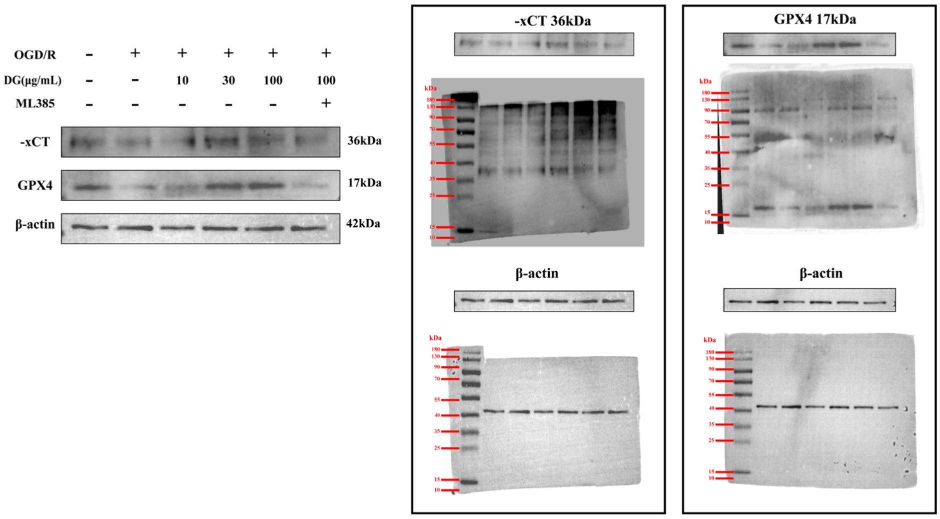

Figure S2. The original image corresponding to Figure 7C in the main text.

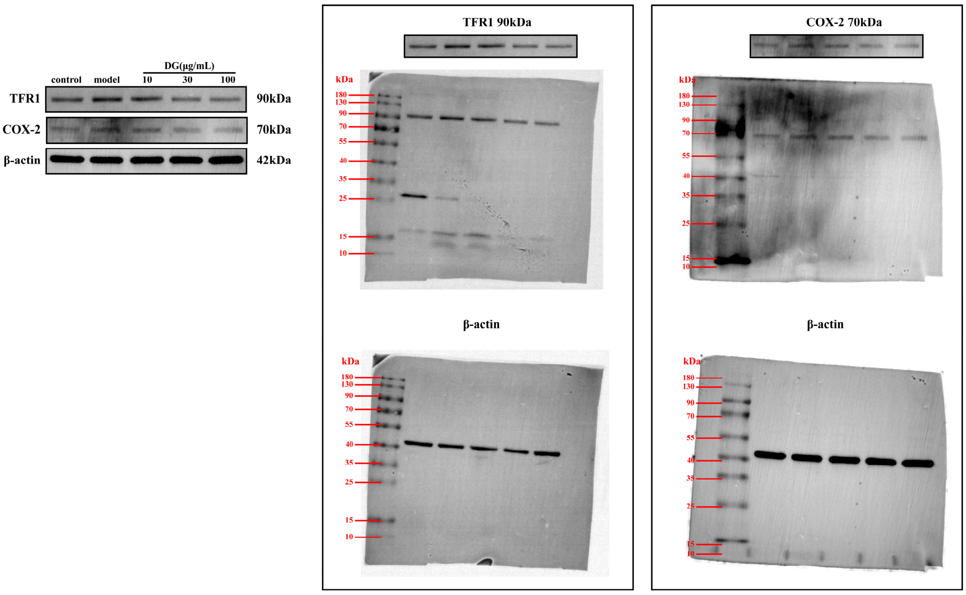

Figure S3. The original image corresponding to Figure 8C in the main text.
